# Safety and Effectiveness of a Novel Tips Access Set with Steerable Cannula in a Swine Model

**DOI:** 10.1007/s00270-023-03544-5

**Published:** 2023-09-18

**Authors:** PengXu Ding, Yujia Ma, Xiaoxia Zhu, Yijie Wu, John Ong, Pu Liu, Jiangqiang Xiao, Yuzheng Zhuge

**Affiliations:** 1 Department of Intervention, First Affiliated Hospital of Zhengzhou University, Zhengzhou, China; 2 Department of Radiology, Shengjing Hospital, China Medical University, Shenyang, China; 3 Medical Affairs, Becton, Dickinson and Company, Shanghai, China; 4 Research & Development, Becton, Dickinson and Company, Shanghai, China; 5 Animal Lab, Shanghai Harborside Medical Technology Co.,Ltd, Shanghai, China; 6 Department of Gastroenterology, Nanjing Drum Tower Hospital, The Affiliated Hospital of Nanjing University Medical School, 321 Zhongshan Road, Gulou District, Nanjing, 210008 China

**Keywords:** Transjugular intrahepatic portosystemic shunt (TIPS), Portal hypertension, Safety, Effectiveness

## Abstract

**Purpose:**

This study aimed to assess the safety, effectiveness, and feasibility of the Liverty™ transjugular intrahepatic portosystemic shunt (TIPS) access set, which has an ergonomic handle that allows for *in situ* cannula tip deflection and a distal steerable cannula angle, *versus* the COOK® Rosch-Uchida Transjugular Liver Access Set (RUPS-100) in healthy pigs.

**Methods:**

Twelve pigs randomly underwent TIPS with the Liverty™ set or the RUPS-100 set. Three interventionalists performed 4 TIPS procedures, 2 with each set. The primary outcome was procedural success, defined as successful establishment of the intrahepatic portosystemic shunt and stent placement.

**Results:**

The shunt was successfully established in 11 pigs. The procedural success was achieved in all 6 pigs in the Liverty™ group and 5 out of 6 pigs for the RUPS-100 group (Fisher exact test, *P* > 0.999). The mean duration of puncture was shorter in the Liverty™ group *versus* the RUPS-100 group (12.3 ± 4.5 min *vs.* 16.2 ± 8.5 min), but without significant statistical difference (two sample* t* test, *P* = 0.359). The cannula angle was adjusted 69% of passes in the Liverty™ group, which was significantly higher than that in the RUPS-100 group (12%, *P* = 0.004). Overall, the TIPS procedural performance was comparable between the groups. Both sets were safe. No intraabdominal hemorrhage, vascular injuries, tissue or organ injuries, porto-biliary fistula, biliary peritonitis, and infection or abscess occurred in either group.

**Conclusion:**

The Liverty™ set is safe and has similar procedural metrics to the COOK® RUPS-100 set. It allows *in situ* adjustment of the angle of the stiffening cannula without increasing procedure time and lessens the occurrences of periprocedural complications.

**Graphical Abstract:**

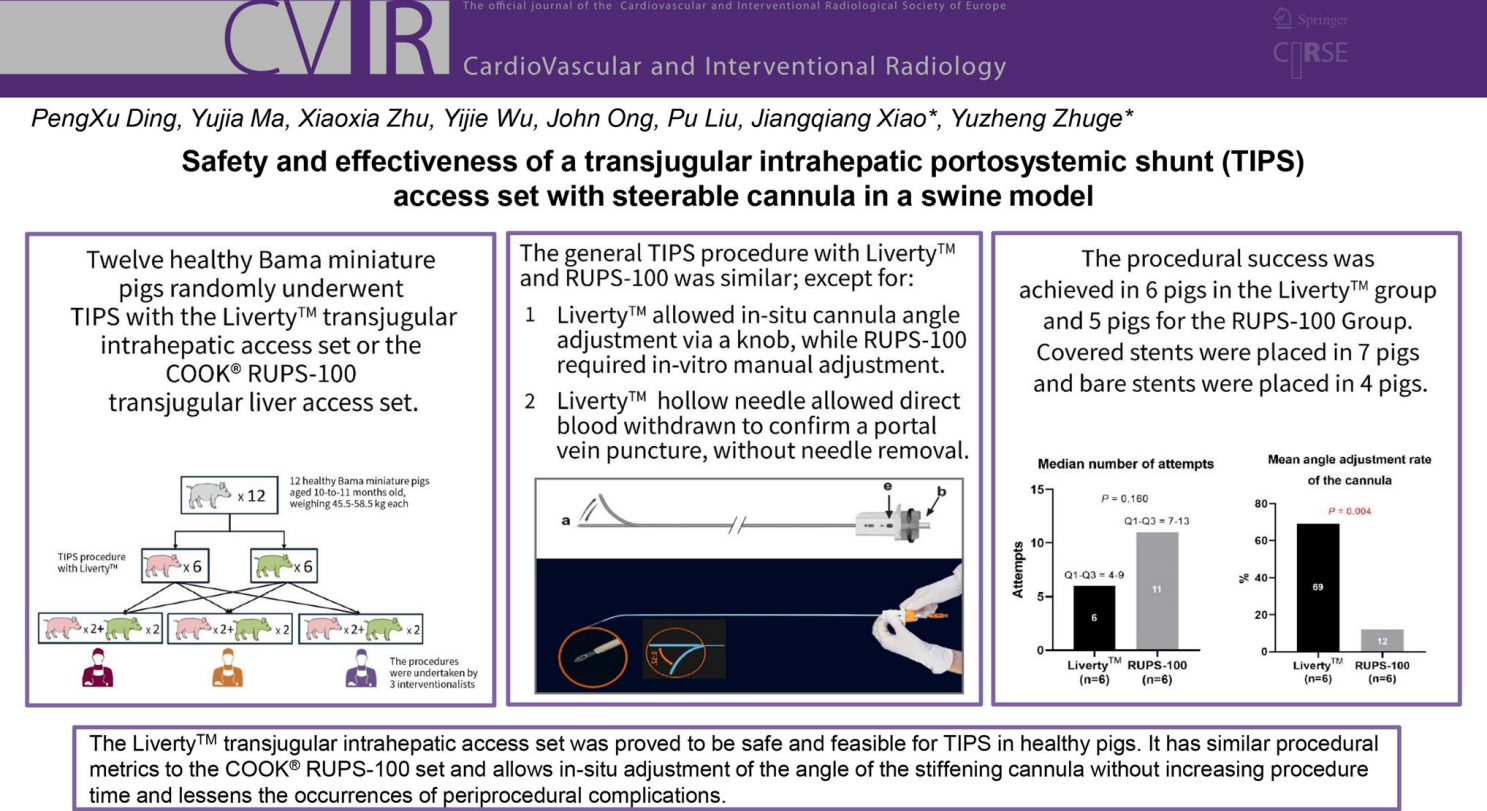

## Introduction

The creation of a transjugular intrahepatic portosystemic shunt (TIPS) is a technically challenging endovascular procedure. Particularly, direct cannulation of the portal branch from the hepatic venous branch requires essentially multiple passes of the needle through the liver, especially in navigating complex portal venous anatomy, despite intra-procedural image guidance using intravenous ultrasound (IVUS) and other modalities like 3-dimensional (3D) image overlay, indirect portography with CO_2_ [1–4]. It usually takes several attempts for the interventionalists to complete the puncture from the hepatic vein to the portal vein with currently available TIPS access sets due to the significant heterogeneity of liver volume and morphology in different patients, or distorted anatomy due to prior TIPS [5]. The stiffening cannula and needle/trocar stylet have to be fully withdrawn for cannula angle adjustment and portal vein confirmation, especially when TIPS is performed involving blind access into a portal vein branch from a hepatic vein in resource-limited settings where intra-procedural image guidance is lacking.

**Fig. 1:**
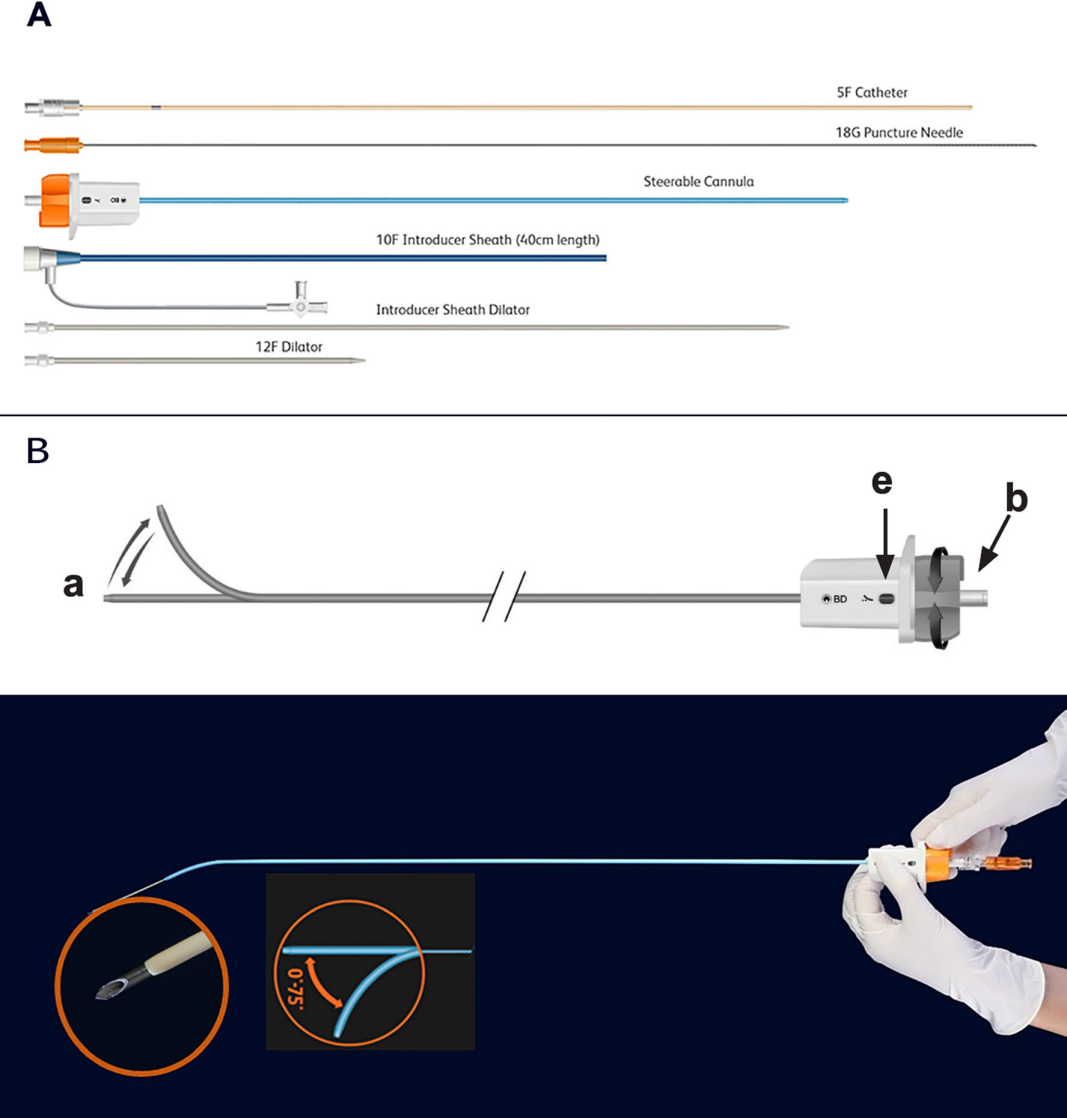
**A** Illustration of the Liverty™ transjugular intrahepatic access set which includes an 18G puncture needle, a 12F dilator, a 10F introducer sheath, an introducer sheath dilator, a Steerable Cannula, and a 5F catheter. **B** The tip (*a*, upper panel) of the distal end of the Liverty™ steerable cannula can be adjusted in situ by the operator by turning the knob (*b*) in the hub (*e*) clockwise to increase or counterclockwise to decrease the angle, with angle adjustment between 0 and 75 degrees. The needle is hollow by design. The illustration in the upper panel is adapted from the BD Liverty™ TIPS access set instructions for use

The BD Liverty™ TIPS Access Set (Fig. 1A) is designed to facilitate the most challenging and risky part of a TIPS procedure, which is to access the portal vein by reducing procedure steps during portal vein puncture. It has an ergonomic handle that allows for *in-situ* cannula tip deflection. Furthermore, the distal end of the steerable cannula can be adjusted externally in incremental and controlled steps by the operator via a knob without removing the cannula from the body, allowing accommodation of individual patient anatomy. In addition, the successful puncture of the portal vein can be confirmed directly through the 18G beveled edge, hollow needle without taking it out (Fig. 1B). Another feature of this product is the hollow needle which allows blood to be directly withdrawn after each puncture, with no need to remove the needle. Moreover, the minor distance (1 mm) between the end of the needle bevel and the tip of 5Fr catheter avoids the loss of the portal vein access before introducing the guidewire.

We speculate that the Liverty™ TIPS Access Set is more effective and safer than the COOK® RUPS-100 transjugular liver access set for cannulation of the portal vein. This randomized study aimed to assess the safety, effectiveness, and feasibility of Liverty™ transjugular intrahepatic access set *versus* the COOK® Rosch-Uchida Transjugular Liver Access Set (RUPS-100) in healthy Bama miniature pigs.

## Materials and Methods

### Animals

This prospective, randomized, controlled, noninferior study was conducted in 12 healthy Bama miniature pigs. Ten-to-eleven-month-old Bama miniature pigs weighing 45.5–58.5 kg each were purchased from Wujiang Tianyu Biological Technology Co., Ltd., and allowed a 7-day adaptation before any study procedure. The animals were maintained in the animal facility with an ambient temperature of 18.9–25.4 °C and a relative humidity of 53.9–69.9%, and a 12 h/12 h light dark cycle.

Procedures involving animals were approved by the local institutional animal care and use committee, and followed the *Guide for the Care and Use of Laboratory Animals* and complied with the *Laboratory Animal Management Principles of China*.

### Techniques

The anatomic structures of the hepatic vein and the portal vein were interrogated based on preoperative CT assessment using a GE Revolution 64 row CT scanner. Ceftriaxone 20–80 mg/kg and tolfenamic acid 2–4 mg/kg for analgesia were given on the day of the procedure, and aspirin 100 mg and clopidogrel were given for 2 to 4 days prior to and on the day of the procedure.

Twelve animals randomly underwent TIPS with the Liverty™ transjugular intrahepatic access set (Becton, Dickinson and Co., Suzhou, China) or the COOK® RUPS-100 transjugular liver access set [6] (COOK, Bloomington, IN, USA). The general TIPS procedure with Liverty™ and RUPS-100 was similar, differing only in the way the cannula tip angle was adjusted and portal vein confirmation (Table 1). The procedures were undertaken by 3 interventionalists who had performed at least 50 TIPS procedures, including 10 TIPS procedures with the COOK® RUPS-100 transjugular intrahepatic access set within the preceding 6 months.

**Table 1:** TIPS procedure with Liverty™ and RUPS-100

TIPS procedure with Liverty™	TIPS procedure with RUPS-100
After anesthesia induction, the right internal jugular vein was surgical exposed, and then, vascular access was established through visual puncture and gradual dilatation. The right or middle hepatic vein was catheterized	
Insert and advance the steerable cannula into the hepatic vein branch. Turn the orange knob to adjust the cannula tip to the desired angle for puncture	Adjust the angle of the stiffening cannula manually. Insert and advance the stiffening cannula into the hepatic vein branch
Thrust the needle/catheter assembly forward through the hepatic parenchyma toward the portal vein	
If the portal vein is not attained: withdraw catheter/needle assembly about 10 cm, rotate the hub to adjust the tip angle of the cannula in situ and re-insert the catheter/needle assembly Connect a syringe to the Luer Lock at the needle hub, apply negative pressure and withdraw the needle / catheter assembly until blood is seen. Confirm portal vein access by injecting contrast medium. Remove the needle from the cannula	If the portal vein is not attained: withdraw the cannula/catheter/needle out of the sheath, bend the tip of the cannula to the desired angle and re-insert the catheter/needle assembly Remove the needle from the cannula, connect a syringe to the Luer Lock at the catheter hub, apply negative pressure and withdraw the catheter assembly until blood is seen. Confirm portal vein access by injecting contrast medium
Introduce a guidewire through the 5F catheter into the portal branch and select the main portal vein. Balloon dilation of the hepatic parenchymal tract and stent placement	

### Study outcomes

The day of surgery was set as Day 0 and imaging study was performed to observe the status of stents on Day 7 ± (1) and the pigs were euthanized for gross anatomical study. Venous blood samples were collected 5 days before surgery and 3 days before euthanasia. Tissue specimens for pathological study were obtained after euthanasia.

The primary outcome of the study was procedural success, defined as successful establishment of the intrahepatic portosystemic shunt and stent placement. Secondary outcomes included the duration of puncture, defined as the interval from the entry of the flexible stiffening cannula or stiffening cannula into the hepatic vein on the first attempt to the time when the wire guide reached the portal vein, and the number of attempted punctures. In addition, the number of adjustments of the angle of the flexible stiffening cannula to orient the tip toward the puncture site was recorded, and the angle adjustment rate was calculated by dividing the number of adjustments of the angle of the flexible stiffening cannula by the number of punctures. The mean volume of injected contrast medium was recorded.

Safety outcomes included the occurrence of intraabdominal hemorrhage, wide variations (> 20% change) in heart rate during the passage of the flexible stiffening cannula or the stiffening cannula through the superior and inferior vena cava, cardiac arrhythmia, and the occurrence of bile tract injuries, porto-biliary fistula, stent migration, post-puncture infection, or biliary peritonitis.

In addition, TIPS procedural performance was evaluated by the interventionalists. A scale of 1 to 5 was used to grade the usability of the operation steps or relevant components, with 1 being very unsatisfactory and 5 being very satisfactory.

### Statistical analysis

Data were expressed in mean and SD, or median and range. Statistical analysis was done using MiniTab (Minitab, LLC, Version: 20.1.3). Normally distributed data were compared using 2-sample *t* test and non-normally distributed data were compared using Mann–Whitney test. Procedural success rate of the two groups was compared using Fisher’s exact test.* P* values less than 0.05 were considered statistically significant.

## Results

### Operative Characteristics and Study Outcomes

In total, 12 TIPS procedures were undertaken, with each of the 3 interventionalists performing two procedures using either the LivertyTM or COOK® RUPS-100 transjugular liver access set. The flexible stiffening cannula or stiffening cannula entered the hepatic vein via the internal jugular vein without any difficulty and the intrahepatic portosystemic shunt was successfully established in 11 pigs. The procedural success was achieved in 6 pigs in the Liverty™ group and 5 pigs for the RUPS-100 Group (Fisher exact test, *P* > 0.999) (Table 2). The mean duration of puncture was shorter in the Liverty™ group *versus* the RUPS-100 group (12.3 ± 4.5 min, Q1,Q3 10,15 *vs*. 16.2 ± 8.5min, Q1,Q3 10–23), but with no significant statistical difference (two sample* t* test, *P* = 0.359). The median number of attempts was 6 (Q1,Q3 4,9) in the Liverty™ group and 11 (Q1,Q3 7,13) in the RUPS-100 group (two sample *t* test, *P* = 0.160). The mean volume of injected contrast medium was 54.2 ± 13.9 mL in the Liverty™ group and was comparable to that of the RUPS-100 group (54.6 ± 12.4 mL; *P* = 0.958). The mean angle adjustment rate of the cannula was 69% (Q1,Q3 56.0%,100.0%) in the Liverty™ group and was significantly higher than that of the RUPS-100 group (12%, Q1,Q3 9%,15%; *P* = 0.004). Covered stents were placed in 7 pigs and bare stents were placed in 4 pigs.

**Table 2:** Study outcomes

Parameters	Liverty™ (n = 6)	RUPS-100 (n = 6)	*P*
Procedural success, n (%)	6	5	> 0.999*
Number of attempts			0.160^
Mean ± SD	5.8 ± 3.3	10.4 ± 6.4	
Median(Q1,Q3)	6(4, 9)	11(7, 13)	
Duration of puncture, min			0.359^
Mean ± SD	12.3 ± 4.5	16.2 ± 8.5	
Median(Q1,Q3)	13(10, 15)	18(10, 23)	
Volume of injected contrast medium, mL			0.958
Mean ± SD	54.2 ± 13.9	54.6 ± 12.4	
Median(Q1,Q3)	56(43, 63)	54(53, 55)	
Angle adjustment rate (%)			0.004
Mean ± SD	69 ± 29	12 ± 8	
Median(Q1,Q3)	65(56, 100)	14(9, 15)	

^*^Fisher exact test; ^two sample t test

### Interventionalists-Assessed Tips Procedural Performance

TIPS procedural performance was assessed by the interventionalists, and the scores for each category are shown in Table 3. Overall, the TIPS procedural performance was comparable between the two groups in the 10 Fr catheter and introducer sheath assembly entering the vena cava, visibility of the tip of the introducer sheath under fluoroscopy, depth and visibility of the trocar stylet/needle under fluoroscopy, and compatibility between the introducer sheath and stent deployment system. The Liverty™ group had a numerically higher score in adequate support by the flexible stiffening cannula during puncture (4.3 ± 0.5 *vs.* 3.7 ± 1.4), the trocar stylet/needle advancing over the parenchymal tract to enter the portal vein (4.3 ± 0.5 *vs.* 4.0 ± 0.6), and the flexible stiffening cannula or stiffening cannula and the introducer sheath reaching the portal vein through the parenchymal tract over the 5 Fr introducer sheath (4.3 ± 0.5 *vs.* 4.0 ± 0.6) than the RUPS-100 group.

**Table 3:** Interventionalists-assessed TIPS procedural performance

Items	Liverty™ (n = 6)	RUPS-100 (n = 6)
The 10 Fr catheter/introducer sheath assembly entering the vena cava		
Mean ± SD	4.3 ± 0.5	4.3 ± 0.5
Visibility of the tip of the introducer sheath under fluoroscopy		
Mean ± SD	4.3 ± 0.5	4.3 ± 0.5
Adequate support by the flexible stiffening cannula during puncture		
Mean ± SD	4.3 ± 0.5	3.7 ± 1.4
The trocar stylet/needle advancing over the parenchymal tract to enter the portal vein		
Mean ± SD	4.3 ± 0.5	4.0 ± 0.6
Depth and visibility of the trocar stylet/needle under fluoroscopy		
Mean ± SD	4.3 ± 0.5	4.3 ± 0.5
The flexible stiffening cannula or stiffening cannula and the introducer sheath reaching the portal vein through the parenchymal tract over the 5 Fr introducer sheath		
Mean ± SD	4.3 ± 0.5	4.0 ± 0.6
Compatibility between the introducer sheath and the stent conveyer system		
Mean ± SD	4.3 ± 0.5	4.3 ± 0.5

1 indicates very unsatisfactory and 5 very satisfactory in each item

### Safety

Overall, the TPS procedure was safe. No wide variation in heart rate (> 20%) occurred upon passage of the flexible stiffening cannula or stiffening cannula through the superior and inferior vena cava. No apparent cardiac arrhythmia occurred during the procedure. In addition, no abnormalities were found in autonomous activities, respiratory function, hair growth, and fecal and urine test.

No intraabdominal hemorrhage, vascular injuries, tissue or organ injuries, porto-biliary fistula, biliary peritonitis, and infection or abscess were observed in any of the pigs in either group. Small amount of blood clot was observed in the hepatogastric ligament or the right costophrenic angle in 2 pigs of the Liverty™ group and in 2 pigs of the RUPS-100 group, respectively.

In 1 pig in the RUPS-100 group, the trocar stylet and 5 Fr catheter assembly failed to reach the portal vein on numerous attempts due to variation in portal anatomy and the procedure was abandoned because of hemorrhagic shock as a result of inadvertent intraperitoneal bleeding.

## Discussion

TIPS placement remains a challenging endovascular procedure, particularly portal branch cannulation from the hepatic venous branch despite preoperative planning such as interrogation of the portal anatomy before TIPS as done in the current study. Multiple needle passes are often required [5]. In this study, we used 10-to-11-month-old Bama miniature pigs for the TIPS procedures. The animals at this particular age have a portal anatomy that is similar to humans [7], allowing completion of a TIPS procedure mimicking the scenario in humans. The study findings indicated that the Liverty™ transjugular intrahepatic access set was safe and feasible for TIPS in healthy pigs.

As the Liverty™ set allows in-procedure *in situ* cannula tip deflection, interventionalists tended to adjust puncture angle as needed to accommodate individual anatomy, which explains a lower adjustment rate in the RUPS-100 group. This “micro and repeated” angle adjustments based on real-time image and feel during passing the parenchyma may contribute to procedural success with the Liverty™ set. Currently available devices have a lower rate of angle adjustment as these devices do not require angle adjustment; thus, the device profile prevents the interventionalist from real-time adjustment. Furthermore, the implementation of an *in situ* real-time adjustable cannula makes it feasible to introduce the insertion ultrasound probe technology at the same time. It enables the puncture angle to be pre-set by ultrasound, one-step cannula angle adjustment and stereotactic puncture. Future investigations will be conducted to address whether the Liverty™ set could increase the efficacy of the procedure, lower the risk, and boost the development of the technique.

All three interventionalists in the study indicated that angle *in situ* adjustment and the hollow design provide ease of operation and improving maneuverability. The Liverty™ group had an overall higher score in adequate support by the flexible stiffening cannula during puncture, the trocar stylet/needle advancing over the parenchymal tract to enter the portal vein, and the flexible stiffening cannula or stiffening cannula and the introducer sheath reaching the portal vein through the parenchymal tract over the 5 Fr introducer sheath than the RUPS-100 group. In addition, operator experience is critical in achieving good procedure metrics and avoiding injuries [8]. There is a correlation [9] between complication rates and number of cases performed by the operators individually. In our study, all three interventionalists performed over 100 TIPS procedures independently with the RUPS-100 set, while using the Liverty™ set for the first time. The difference of device familiarity between the two groups may have introduced some bias into the study despite the preoperative introduction of Liverty™ by the engineers and the similarity for most parts of the procedures.

It should be noted that the interventionalists were not blinded to the device being used. In addition, though TIPS can be performed in pigs with the same techniques as in humans [10], the portal venous anatomy in pigs differs from humans. Despite that the interventionalists in the study were experienced in TIPS procedures, they may not be well versed in porcine anatomy, which could lead to inaccurate positioning during TIPS. TIPS procedural performance was evaluated by the interventionalists using a scale that has not been validated, which could lead to potential bias. In the current study, the portal vein was punctured based only on fluoroscopic imaging without any kind of intra-procedural image-guidance. This led to a high number of attempts (6 in the Liverty™ group and 11 in the RUPS-100 group). IVUS and intra-procedural image guidance using other modalities like 3D image overlay, indirect portography with CO_2_ would allow for TIPS being performed with fewer puncture attempts [1–3].

In conclusion, the Liverty™ set is safe and has similar procedural metrics to the COOK® RUPS-100 set. It allows *in situ* adjustment of the angle of the stiffening cannula without increasing procedure time and lessens the occurrences of periprocedural complications. The study findings support further clinical development of the Liverty™ transjugular intrahepatic access set, including clinical applications.

## Abbreviation

TIPS: Transjugular intrahepatic portosystemic shunt

## References

[1] Dastmalchian S, Aryafar H, Tavri S (2022). Intravascular ultrasound guidance for tips procedures: a review. AJR Am J Roentgenol.

[2] Farsad K, Kaufman JA (2016). Novel image guidance techniques for portal vein targeting during transjugular intrahepatic portosystemic shunt creation. Tech Vasc Interv Radiol.

[3] Herren JL, Shah KY, Patel M, Niemeyer MM (2023). Intravascular ultrasound for transjugular intrahepatic portosystemic shunt creation: "TIPS" and Tricks. Semin Intervent Radiol.

[4] Luo X, Wang X, Yu J, Zhu Y, Xi X, Ma H (2018). Transjugular intrahepatic portosystemic shunt creation: three-dimensional roadmap versus CO(2) wedged hepatic venography. Eur Radiol.

[5] Sinha I, Goldman DT, Patel RS, Nowakowski FS (2023). Advanced techniques for accessing the portal vein during transjugular intrahepatic portosystemic shunt creation. Semin Intervent Radiol.

[6] Partovi S, Li X, Shwaiki O, Rashwan B, Ruff C, Grozinger G (2021). Advanced portal venous access techniques for transjugular intrahepatic portosystemic shunt placement. BMJ Open Gastroenterol.

[7] Lada E, Anna M, Patrik M, Zbynek T, Miroslav J, Hynek M (2020). Porcine liver anatomy applied to biomedicine. J Surg Res.

[8] Lopera JE (2023). A comprehensive review of transjugular intrahepatic portosystemic shunt-related complications. Semin Intervent Radiol.

[9] Dariushnia SR, Haskal ZJ, Midia M, Martin LG, Walker TG, Kalva SP (2016). Quality improvement guidelines for transjugular intrahepatic portosystemic shunts. J Vasc Interv Radiol.

[10] Seo TS, Oh JH, Park YK, Song HY, Park SJ, Yuk SH (2005). Efficacy of a dexamethasone-eluting nitinol stent on the inhibition of pseudo-intimal hyperplasia in a transjugular intrahepatic portosystemic shunt: an experimental study in a swine model. Korean J Radiol.

